# Examination of patient flow in a rural health center in Malawi

**DOI:** 10.1186/s13104-016-2144-x

**Published:** 2016-07-25

**Authors:** M. A. Jafry, A. M. Jenny, S. J. Lubinga, E. Larsen-Cooper, J. Crawford, C. Matemba, J. B. Babigumira

**Affiliations:** 1 University of Washington, Seattle, WA USA; 2 Global Medicines Program, University of Washington, Seattle, WA USA; 3 Pharmaceutical Outcomes Research and Policy Program, University of Washington, Seattle, WA USA; 4 VillageReach, Seattle, WA USA; 5 VillageReach, Lilongwe, Malawi

**Keywords:** Health centers, Malawi, Rural, Wait times, Patient satisfaction, Time-motion, Patient flow

## Abstract

**Background:**

Malawi, like many low-income countries, is facing a severe health worker shortage. A potential stop-gap solution to this crisis is improving the efficiency of health center operations. Given the lack of research on center efficiency in rural health centers in Malawi, we conducted a study to identify deficiencies in center organization and barriers to patient flow.

**Methods:**

We performed a time-motion survey at a rural health center in Ntaja, Malawi over a period of 1 week. We used a standardized questionnaire to collect information on the amount of time a patient spent with each health worker, the number of center staff that attended to each patient, and the total time spent at the center. Additionally, at the end of the visit, we conducted an exit survey to collect demographic information and data on perception of quality of care with the center visit for all patients.

**Results:**

A total of 1018 patients were seen over the five-day study. The average total time spent at the center by the patients was 123 min (2–366 min). Adults had an average total time spent at the center of 111 min (2–366 min) and children 134 min (7–365 min). Patient waiting time (PWT) was higher in the early morning hours ranging from 157 min (between 06:00 and 08:00) to 53 min (between 14:00 and 16:00). Health worker contact time (HCT) was higher for adults (2.3 min) than children (1.7 min). Shorter wait times were associated with higher perceptions of quality of service.

**Conclusion:**

Despite shortages in health workers and funds, opportunities are available to increase efficiency in rural health centers. By removing bottlenecks to increase the productivity of health workers, centers in low-income countries can treat more patients and improve service quality.

**Electronic supplementary material:**

The online version of this article (doi:10.1186/s13104-016-2144-x) contains supplementary material, which is available to authorized users.

## Background

Malawi, like other low-income countries, is facing a healthcare crisis due to severe health worker shortages and an overloaded health care system [1]. Ranked 179th out of 194 countries in per capita health expenditure, Malawi spends only $49 per capita annually [2]. Similarly, Malawi only has 0.19 doctors and 2.83 nurses and midwives per 10,000 people, putting the number of doctors in the entire country at 302 [3]. Output at medical teaching institutions in Malawi is too low to fill current vacant positions, let alone expand access to healthcare services [3].

Due to these shortages, most health centers in Malawi do not have doctors and instead rely on lower-level health workers with varied levels of professional training, ranging from 2 years to little or no professional training [4]. For instance, a typical rural health center in Malawi is run by a medical assistant (MA) supported by other health workers including nurse-midwives, both with over a year of professional training, as well registry clerks, drug store clerks, health surveillance assistants (HSAs), and “attendants” who often have limited or no training, and perform support duties and some aspects of clinical care. While these lower-level health workers play an integral role in improving access to healthcare services, their lack of training may lead to inefficiencies in the running of the health center. These difficulties are exacerbated by the low number of health centers in Malawi, leading to the already understaffed centers being overloaded.

Interventions to improve health center operations in Malawi may enable the country to better meet the health needs of its rapidly growing population [5]. As recruiting and training additional health care providers will require substantial time and resources, increasing the efficiency of the health center operations and health care provider interventions—training, task-shifting, health worker re-organization, and health worker re-deployment—may present a viable stop-gap solution.

There is currently a dearth of research studying health center efficiency in Malawi. An understanding of patient flow and center organization is imperative for improving efficiency in health centers. Time-motion studies provide detailed information about the flow of patients through the center as well as identify different bottleneck in the clinical structure. Previous time motion studies conducted in Sub-Saharan African countries have been able to pinpoint significant deficiencies in center organization and barriers to patient flow, such as poor allocation of health provider time and inefficient record management [6–8]. Many of these studies also proposed interventions which, when implemented, were able to greatly reduce waiting times and increase the number of patients treated [9].

In this paper we describe a five-day time-motion study of patient flow at a health center in Ntaja, Malawi to identify bottlenecks and better understand center organization. This study was part of a broader study to measure the impact on access to medicines, health outcomes, and health center operational efficiency of an innovative pharmacy assistant training program and supply chain strengthening in Malawi [10].

## Methods

### Time motion survey

We performed a one-week time-motion survey at a rural health center in Ntaja, Malawi. The health center was surveyed for the entire week, excluding weekends, to capture the variability of services offered and type of care sought at the center throughout the week. All outpatients visiting the center during the week of the survey were invited to participate in the study. Once informed consent was received, each participant was assigned a unique identification number.

We used a standardized questionnaire to collect information on the amount of time a patient spent with a health worker, the number of center staff each patient spent time with during their visit to the health center, the waiting time before patients saw each health worker, and the total time spent at the center. Participants were instructed to carry the questionnaire to each health care worker they saw. Each health worker the patient saw filled out time of entry and exit for themselves, as well as their own initials and staff designation. All health workers were instructed to expect the time sheet and to inquire about it if the patient did not present it.

### Patient experiential survey

At the end of the center visit, participants were interviewed about whether they sought care for themselves or someone else; the symptoms prompting seeking of care; time and money spent while travelling to the center; diagnosis given by health worker; medicines prescribed and whether each prescribed medicine was dispensed; and a rating of quality of service received, ranging from “very bad” to “excellent.”

### Data analysis

We performed descriptive analyses using means and proportions to characterize the patient population. We used means and standard deviations to characterize times (waiting time, health worker contact time, and total time spent at the center) by patient characteristics, time of the day, day of the week and service quality.

## Results

A total of 1018 patients participated in the study over the five-day period.

### Characteristics of respondents

The characteristics of the patients included in the survey are shown in Table 1. A nearly equal proportion of adults and children visited the center (51 vs. 49 %). Monday and Tuesday were the busiest center days with approximately 23 % of center visits each during the week. Fever was the most common presenting symptom occurring in 51 % of patients. Muscular or skeletal pain was the most common diagnosis and was made by clinicians in 30 % of patients. Paracetamol was the most common medicine prescribed (in 30 % of patients) and the most frequently out of stock (it was unavailable to 59 % of patients to whom it was prescribed). Vitamins were also regularly co-prescribed with Paracetamol (68 % of patients prescribed Paracetamol were prescribed vitamins). Overall the vast majority of patients (77 %) who had a medicine prescribed had it dispensed. Most patients rated the quality of clinical services as very good (36 %) or good (34 %).

**Table 1:** Characteristics of patients attending health center in Ntaja, Malawi

Characteristic	Number (%) N = 1018
*Age group*	
Children	518 (50.9 %)
Adults	500 (49.1 %)
*Travel time to center (minutes)*	55.3 (44.9)
*Transport costs to center ($US)*	0.12 (0.33)
*Day of center visit*	
Monday	237 (23.3 %)
Tuesday	231 (22.7 %)
Wednesday	166 (16.3 %)
Thursday	184 (18.0 %)
Friday	201 (19.8 %)
*Presenting symptom*	
Fever	563 (55.2 %)
Cough	397 (38.9 %)
Diarrhea	68 (6.7 %)
Body ache	232 (22.8 %)
Other	439 (43.0 %)
*Diagnosis*	
Muscular/skeletal pain	302 (29.8 %)
Malaria	286 (28.3 %)
Respiratory infection	197 (19.5 %)
Fever	79 (7.8 %)
Other	148 (14.6 %)
*Medicine prescribed*	
Paracetamol	625 (41.2 %)
Multivitamins	394 (26.0 %)
Lumefantrine/artemether	266 (17.5 %)
Fansidar	48 (3.2 %)
Clotrimazole	45 (3.0 %)
Other	139 (9.1 %)
*Medicines prescribed were dispensed*	
Yes	3854 (89.9 %)
No	434 (10.1 %)
*Medicines prescribed but not dispensed*	
Paracetamol	229 (52.8 %)
Multivitamins	82 (18.9 %)
Aspirin	11 (2.6 %)
Oral rehydration salts	10 (2.3 %)
Calamine lotion	10 (2.3 %)
Other	92 (21.2 %)
*Patient satisfaction*	
Excellent	8 (0.8 %)
Very good	234 (23.0 %)
Good	472 (46.3 %)
Poor	245 (24.0 %)
Very poor	60 (5.9 %)
*Number of providers seen*	
One	83 (8.1 %)
Two	244 (23.9 %)
Three	462 (45.3 %)
Four	203 (19.9 %)
Five	27 (2.7 %)
Six	1 (0.1 %)

The number of providers seen by each patient varied considerably; 32 % of patients saw one or two providers, 65 % saw three or four providers, and 3 % saw five or six providers.

### Patient waiting time

Patient waiting time (PWT) is summarized in Table 2. PWT was higher for children (133 min) than adults (108 min), and children saw more providers on average (2.99 providers) than adults (2.72 providers) per visit. PWT was higher on Thursday and Friday (both 136 min) than on other days of the week, and there were fewer providers seeing patients on both of these days (6 and 4 respectively), as compared to Monday, Tuesday and Wednesday (7, 7, and 8 respectively). PWT was higher in the early morning hours ranging from 157 min (between 06:00 and 08:00 h) to 53 min (between 14:00 and 16:00 h). PWT was higher before patients were seen by registry clerks (67 min) and medical assistants (60 min) but shorter before patients were seen by hospital attendants (35 min) and pharmacy attendants (14 min). When PWT was higher, patients generally reported worse quality of clinical services (e.g. average waiting time of 129 min when patients reported poor service quality) quality than when patients reported better clinical service quality (e.g. average waiting time of 92 min when patients reported excellent service quality).

**Table 2:** Waiting time (in minutes), health worker contact time, and overall time spent by patients at health center in Ntaja, Malawi

Characteristic	Waiting time mean (SD)	Contact time mean (SD)	Total time mean (SD)
*Patient type*			
Adult	108.4 (67.6)	2.3 (6.0)	110.7 (67.9)
Children	133.2 (65.4)	1.7 (4.3)	134.9 (65.5)
*Day of the week*			
Monday	121.5 (80.7)	2.2 (5.4)	123.7 (80.8)
Tuesday	109.7 (64.4)	2.2 (8.0)	111.9 (64.6)
Wednesday	98.4 (41.1)	1.0 (3.8)	99.4 (41.1)
Thursday	135.9 (50.0)	2.3 (3.3)	138.2 (50.3)
Friday	136.0 (76.4)	1.8 (3.1)	137.8 (76.6)
*Time of day*			
06:00–08:00	156.6 (58.1)	1.8 (5.3)	158.4 (58.3)
08:00–10:00	133.4 (61.7)	2.0 (3.9)	135.4 (61.8)
10:00–12:00	73.4 (50.8)	1.5 (2.0)	74.9 (51.0)
12:00–14:00	65.6 (35.3)	1.8 (4.3)	67.4 (35.6)
14:00–16:00	52.7 (26.7)	2.0 (5.0)	54.7 (27.5)
*Healthcare cadre*			
Registry clerk	68.8 (55.3)	0.6 (3.0)	69.4 (55.3)
Hospital attendant	34.7 (34.5)	1.2 (6.0)	35.9 (34.6)
Medical assistant	59.9 (52.7)	1.1 (3.7)	61.0 (52.8)
Pharmacy attendant	13.5 (20.7)	0.4 (2.7)	13.9 (20.7)
*Patient satisfaction*			
Excellent	92 (71.0)	1.9 (2.5)	93.9 (71.3)
Very good	110.2 (66.1)	1.7 (5.1)	111.9 (66.2)
Good	122.0 (68.7)	1.8 (4.3)	123.8 (68.8)
Poor	129.0 (63.1)	1.8 (5.0)	130.8 (63.3)
Very poor	121.0 (75.9)	2.6 (4.0)	123.6 (76.2)
*Overall*	120.6 (67.5)	2.0 (6.4)	122.6 (67.6)

### Health worker contact time

Health worker contact time (HCT) is summarized in Table 2. HCT was higher for adults (2.3 min) than children (1.7 min). HCT was substantially lower on Wednesday (1.0 min) than on other days (2.2. minutes (Monday and Tuesday), 2.3 min (Thursday) and 1.8 min (Friday). HCT was lowest between 10:00 h and 12:00 h but generally similar at different times of the day. HCT was lowest for pharmacy attendants (0.4 min) and highest for hospital attendants (1.2 min). HCT was not generally related to reported service quality ranging from 1.8 min when patients reported good or poor service quality to 2.6 min when patients reported very poor service quality.

### Total time spent at the center

The total time spent in the center is summarized in Table 2. The mean total time spent at the center was 123 min. The total time spent in the center was higher for children (135 min), on Thursdays (138 min), early in the morning (158 min between 06:00 and 08:00), and among patients who rated the quality of clinical services as poor (131 min).

## Discussion

In this time motion study, we demonstrate that patients at a rural health center in Malawi spend a substantial amount of time at the center (123 min) and that a large proportion of the time (121 min) or (98 %) is spent waiting for services either at the beginning of the center visit (before seeing a registry clerk) or between health seeing different health workers. The longest waiting times appeared to be before seeing registry clerks (69 min) and before seeing medical assistants (60 min) indicating bottlenecks in service delivery.

From our discussion with the health workers at the center, we expected to find a general order of health workers seen by the patient to start with the registry clerk, then move to either the medical assistant or the hospital attendant, and finally go to the pharmacy attendant. However, our results showed that this was not the case and no such order was present.

Our data suggest that most patients in rural Malawi (65 % in this study) arrive at centers before 10:00 a.m. This distribution of out-patient visit times causes the center’s capabilities to be overloaded in the morning and underused in the afternoons; the total waiting time and total time at the center for patients arriving before 10:00 a.m. was approximately two and a half times longer than for patients arriving after 10:00 a.m. Other patient flow studies in Uganda and Nigeria have found similarly longer waiting times earlier in the morning than later in the day [8, 11]. This bottleneck could potentially be removed by redistribution of patient arrivals throughout the day. As the center in Ntaja is a walk-in center, no appointments are given and patients arrive at the times they choose. If an information drive is introduced to inform patients in the area about the benefits of arriving after 10:00 a.m., the center could ameliorate the backlog of patients in the mornings and reduce waiting times.

A large proportion of the waiting time for patients was spent waiting to see the registry clerk, similar to results found in a patient-flow study in Uganda [7]. These large waiting times imply a need to improve and optimize the health record management system [7]. This optimization can be done through better organization of the record management system, as well as with computerization of the registry, depending on resource availability. Both of these techniques have been shown to be effective in low-resource settings [12]. Another potential intervention includes better training for the registry clerks or increasing the number of clerks, interventions that have been efficacious when applied in other centers [13].

The variation in average total time spent at the center between days of the week did not have a significant correlation with the number of patients seen on that day, indicating other factors are also influencing the average total time. Similar time-motion studies in Uganda have pointed to poor allocation of health providers’ time as causing long visits and wait times, which may be contributing to this variation in average total time [6].

Low quality of care perception has been shown to be an issue in most resource-constrained health centers by many studies in Nigeria, Tanzania, Kenya, and Ghana [11, 14–16]. The main factors leading to the perception of poor perception of quality of care in these studies was predominantly long waiting times and drug stock outs [14, 16]. Our results showed a similar correlation as patients who rated the quality of care as poor or very poor, had longer total center times than patients who rated the quality as good, very good, or excellent. By reducing waiting time and better managing drug stocks, the health center could significantly increase health center service quality and quality of care perception.

The center also experienced a drug shortage of Paracetamol during our five-day study. Twenty-seven percent of the patients did not receive one or more of the drugs they were prescribed, with the majority not receiving Paracetamol (59 %). There may have been awareness among center staff about the availability of medicines on a given day as prescription patterns varied based on medicine availability in the pharmacy. For example, of the patients prescribed Paracetamol, 338 (68 %) of them were also prescribed vitamins, and the vitamins were only dispensed when Paracetamol was not available, indicating vitamins may have been often prescribed as a substitute for Paracetamol at times when Paracetamol stock was low. Additionally, there was a rationing of Paracetamol evident in our data over all 5 days, as patients in the morning were able to receive Paracetamol while patients in the afternoon were more likely to get vitamins. As drug stock management is a complex task requiring training and experience to do properly, the drug store clerks could potentially benefit from receiving more specialized training regarding pharmacy management and drug stock evaluation [17]. The three most common diagnoses for patients prescribed Paracetamol were in descending order: muscular skeletal pain, respiratory infection, and fever.

We found disorganization in the sequence of health workers seen by the patients at this health center. There were neither defined triage nor check-in personnel. Which health worker triaged the patient had no significant correlation with the symptoms reported nor the diagnosis the patients received. This lack of defined triage personnel is potentially a large contributor to the long wait times. Efficient triage can cut down on the number of health providers seen by patients by directing patients to the needed provider. Plus, more organized management of check-ins can likely help reduce the backlog of patients in the morning by efficiently utilizing all the health providers, circumventing other bottlenecks. Studies in Sub-Saharan Africa and in resource-constrained settings have shown how well organized triage can increase patient and staff satisfaction [1, 13, 17–20]. Similar studies have also demonstrated that low-level health workers in resource-limited settings, with proper training, can reliably perform triage [21].

In line with these recommendations, a program is currently being implemented to train, deploy, and support an enhanced cadre of Pharmacy Assistants in low-resource health centers in Malawi. This three-year initiative, currently in progress, will train and deploy facility-based pharmacy staff, provide supply chain capacity building to multiple districts in Malawi, and improve reporting of logistical data. Additionally, this program will also serve as a model for other low- and middle-income countries (LMICs) for improving care at their low-resource health centers.

A weakness in this study that deserves mention is our use of a health provider-filled questionnaire instead of a researcher-filled questionnaire, creating the possibility of inaccurate time reporting and inconsistencies in how times were recorded. On the other hand, there are strengths in the study that also deserve mention: (1) there was a large sample size; (2) the study was done over a period of 5 days and so we were better able to measure variations and patterns over an entire week; and (3) the questionnaire recorded initials of the health providers instead of just their titles, allowing us to pinpoint any discrepancies in time recording as well as to better understand variations between providers.

## Conclusion

This study highlights challenges faced at a rural center in Malawi. Patients often travel long-distances to seek medical care when they or their child are ill and on average wait nearly 2 h for a few minutes of interaction with a healthcare provider. Over 10 % of the time, these patients leave the center without the medicines prescribed for treatment. By removing bottlenecks, health centers can reduce wait times and potentially treat more patients. Additionally, having dedicated personnel such as a trained, certified pharmacy workers can improve drug stock management and reduce stock outs.

## Additional file

10.1186/s13104-016-2144-x Time motion data.

## Abbreviations

PWT: total patient waiting time
HCT: total health worker contact time
MA: medical assistant
HSA: Hospital Surveillance Assistant
